# Obsessive–compulsive personality disorder in rapid eye movement sleep behavior disorder

**DOI:** 10.1038/s41598-022-06424-z

**Published:** 2022-02-14

**Authors:** Antonina Luca, Calogero Edoardo Cicero, Loretta Giuliano, Riccardo Sgroi, Edoardo Vancheri, Claudio Terravecchia, Raffaele Squillaci, Cristina Rascunà, Giulia Donzuso, Giovanni Mostile, Giorgia Sciacca, Mario Zappia, Alessandra Nicoletti

**Affiliations:** Neurologic Unit, AOU “Policlinico-San Marco”, Department of Medical, Surgical Sciences and Advanced Technologies, GF Ingrassia, University of Catania, Via Santa Sofia n.78, 95100 Catania, Sicily Italy

**Keywords:** Parkinson's disease, Sleep disorders

## Abstract

Rapid eye movement sleep behavior disorder (RBD) is a common prodromic non-motor symptom of Parkinson’s disease (PD). Only few studies have evaluated the personality of RBD patients with conflicting results. Aim of the study was to evaluate the frequency of Personality Disorders (PeDs)in RBD. RBD patients, PD patients and healthy controls (HC) were enrolled. All the enrolled subjects underwent a full neurological examination. Motor symptoms were evaluated with the UPDRS-Motor Examination. PeDs were assessed with the Structured Clinical Interview for DSM-IV Personality Disorders (SCID-II). Twenty-nine RBD patients [14 men (48.3%); mean age 55.6 ± 11.1], 30 PD patients [17 men (56.7%); mean age 65.7 ± 10.7] and 30 HC [12 men (40%); mean age 65.7 ± 5.4] were enrolled in the study. PD patients had a disease duration of 4.5 ± 4.6 and presented a mean UPDRS-ME score of 26.7 ± 9.4. The most frequent PeDs was the Obsessive–Compulsive one (OCPeD); OCPeD was significantly more frequent in RBD (55.2%) patients than HC (13.3%; *p*-value < 0.001). No significant differences were found comparing the frequency of OCPeD in RBD patients to that in PD. In the present study, the prevalence of OCPeD in RBD patients was close to that reported in PD patients. Our data could suggest the existence of a common disease-specific RBD-PD personality profile.

## Introduction

Rapid eye movement (REM) sleep behavior disorder (RBD) is a parasomnia characterized by dream contents enactment and excessive motor activity, such as punching, gesturing, crying and kicking, due to the loss of the physiological REM sleep muscle atonia^1^. Even if the exact prevalence of RBD is still unclear, a recent metanalysis reported that the pooled estimate prevalence of probable RBD (pRBD, without a polysomnographic recording confirmation) is 5.65%^2^. According to the International Classification of Sleep Disorders, RBD can be classified in “symptomatic”, accompanying some neurodegenerative disorders, brain lesions, autoimmune disorders or drugs, and “idiopathic” RBD (iRBD)^1^. Notably, longitudinal studies have demonstrated a strong association between the latter and the occurrence of synucleinopathies, such as Parkinson’s disease (PD), Multiple System Atrophy, Lewy Bodies Dementia, with a risk of *phenoconversion* increasing proportionally to follow-up duration. On the other hand, from 46 to 80% of patients with synucleinopathy have RBD and, in almost all disorders, it has anticipated by several years the onset of motor symptoms. Actually, RBD has been so consistently associated with the presence of an underlying synucleinopathy that is now probably considered the strongest non-motor symptom predictor of PD, representing an early warning^3^. Hence, in the perspective of an early diagnosis and treatment, the interest in the assessment of potential prodromal markers of PD such as constipation, hyposmia, cognitive impairment and personality changes in iRBD patients is growing.

Interestingly, some neuropsychiatric disorders, including mood disorders^4^, attention-deficit/hyperactivity disorder^5^, and personality characteristics^6^, have been associated with risk of PD occurrence. Concerning personality, since earlier observations, parkinsonian patients have been described as extremely industrious, introvert, rigid, punctual, moralistic. More recently, parkinsonian personality has been evaluated according to different models of personality assessment^7^. In particular, as reported by a recent metanalysis, studies based on the Big Five Model (BFM) of personality described parkinsonian personality as characterized by high Neuroticism and low Openness and Extraversion, while studies based on the Cloninger's Psychobiological Model (CPM) described it as characterized by low Novelty Seeking (NS) and high Harm Avoidance (HA) levels^8^. Finally, studies applying the Diagnostic and Statistical Manual for Mental Disorders (DSM) criteria, reported that more than 40% of PD patients had Obsessive–Compulsive Personality Disorder (OCPeD), even at the time of PD diagnosis^9^.

Although the existence of a parkinsonian personality has been demonstrated over the years, nowadays only few studies have evaluated whether the personality of iRBD patients presents some “similarities” with the parkinsonian one^10^. Moreover, these studies reported conflicting results and never evaluated the presence of Personality Disorders (PeDs). Aim of the study was to assess the presence of PeDs in patients with iRBD compared to a group of patients suffering from PD and Healthy Controls (HC).

## Methods

### Study population

Three groups of subjects were enrolled: iRBD patients, PD patients and Healthy Controls (HCs).

iRBD were enrolled among subjects previously identified in a population-based study investigating iRBD prevalence in the communality of Catania^11^ and among outpatients attending the Neurologic Clinic of the University of Catania for a sleep complain.

The diagnosis of iRBD was made according to the diagnostic criteria proposed by the American Academy of Sleep Medicine^1^. Diagnosis of definitive iRBD (dRBD) was sought on the base of a video-polysomnographic recording (VPSG), showing the lack of the physiological atonia during REM sleep phase associated to abnormal motor behaviors or vocalizations; when VPSG was not available, after a complete examination performed by a board-certified sleep expert (LG), the diagnosis of probable iRBD (pRBD) was made^1^.

The diagnosis of clinically established PD was made according to the Movement Disorders Society diagnostic criteria^12^. HCs were consecutively recruited among patient’s caregivers or accompanying. They underwent a clinical interview and a standard neurological examination. HC with probable RBD (positive at the single question questionnaire and/or symptoms reported by partner) and neuropsychiatric disturbances were excluded from the study.

All the enrolled subjects (RBD, PD and HCs) underwent the Mini Mental State Examination (MMSE)^13^ and a clinical interview. Subjects with a MMSE < 24, impairment of activities of daily living and/or psychiatric disorders (i.e. depression, anxiety, panic attack, psychosis, drugs utilization, alcoholism…) were excluded from the study.

### Clinical evaluation

All the enrolled subjects underwent a full neurological examination. PD patients were clinically evaluated when in “Off” state. Motor symptoms were evaluated with the Unified Parkinson’s Disease Rating Scale-Motor Examination (UPDRS-ME)^14^ Questionnaires and clinical interview were performed when in “On” state. To assess the presence of OCPeD we adopted the widely used Structured Clinical Interview for Diagnostic and Statistical Manual for Mental Disorders-Axis II Personality Disorders (SCID-II) and the associated SCID-II Personality Questionnaire (SCID-II-PQ)^15^. In the typical use of the SCID-II assessment approach, the subject completes a self-report questionnaire (the SCID-II-PQ) then, to confirm the diagnosis of personality disorders, only positive items are further explored in a semi-structured and diagnostic interview (the SCID-II) performed by a neurology with an expertise in neuropsychology (AL).

### Ethics

The study has been approved by the ethical committee of the University Hospital “Policlinico-San Marco” of Catania. All methods were performed in accordance with relevant guidelines and regulations. All the enrolled subjects signed an informed consent prior to be included in the study.

### Statistical analysis

Data were analysed using STATA 16.0 software packages. Quantitative variables were described using mean and standard deviation. The difference between means and the difference between proportions was evaluated by the t-test and the Chi squared test respectively. In case of not a normal distribution appropriate non-parametric tests were performed. Univariate analysis, logistic regression was performed in order to evaluate possible associations between clinical variables and RBD (outcome variable). Multivariate analysis, adjusted by age and sex considered a priori confounders, was performed.

## Results

Twenty-nine iRBD patients (14 men; age 55.6 ± 11.1 years), 30 PD patients (17 men; age 65.7 ± 10.7 years) and 30 HC (12 men; age 65.7 ± 5.4 years) were enrolled in the study. Subjects with iRBD were significantly younger than HC (*p*-value < 0.0001) and PD patients (*p*-value < 0.001).

Out of 29 subjects with iRBD, 11 (37.9%) were dRBD (6 men, age 56.1 ± 14.1 years) and 18 (62.1%) were pRBD (8 men, age 55.3 ± 9.0 years). No statistically significant differences were found when comparing demographic characteristics of dRBD to pRBD. PD patients had a disease duration of 4.5 ± 4.6 years and presented a mean UPDRS-ME score of 26.7 ± 9.4. Eighteen (60%) PD patients were never treated and 8 (26.7%) had pRBD.

OCPeD was the most frequent personality disorder, being recorded in 16 (55.2%) subjects with iRBD, 14 (46.7%) PD patients and 4 (13.3%) HC. Concerning others PeDs, among iRBD subjects, 3 (10.3%) had avoidant PeD and 1 (3.4%) had dependant PeD while, among PD patients, depressive PeD was recorded in 3 (10%) and paranoid PeD in 1 (3.3%). In the HC group, 1 (3.3%) individual had avoidant PeD and 1 (3.3%) had depressive PeD.

OCPeD was significantly more frequent in iRBD than in HC (*p*-value 0.001). No significant differences were found comparing the frequency of OCPeD in iRBD and in PD (*p*-value 0.514). OCPeD was recorded in 10 (45.4%) out of the 22 PD patients without pRBD and 4 (50%) out of the 8 PD patients with pRBD. No statistically significant difference was recorded in OCPeD frequency comparing PD patients with pRBD and without pRBD (*p*-value 0.825).

At univariate analysis OCPeD was significantly associated with iRBD (outcome variable) (OR 6.7; 95%CI 2.08–21.66; *p*-value 0.001). The association was confirmed at multivariate analysis adjusting for age and sex, considered a priori confounders (OR 6.3; 95%CI 1.88–21.42; *p*-value 0.003).

A formal statistical comparison analysis considering the other PeDs was not performed due to the small number of patients in the three groups.

## Discussion

Obsessive compulsive personality disorder is probably the most frequent personality disorder in the general population and is characterized by the presence of rigid perfectionism, perseveration, restricted affectivity and intimacy avoidance.

Confirming our previous finding^9^ we recorded a higher prevalence of OCPeD among PD patients (46.7%) than HC (13.3%). Interestingly, in iRBD patients the prevalence of OCPeD was very close to that reported in PD patients (55.2%). Moreover, a strong association between RBD and OCPeD was found (OR 6.7). Concerning the others PeDs, as previously reported^9^, the depressive PeD was the second most frequent one, at least among PD patients, while the others PeDs, less common also in the general population, were minimally or not represented at all. Probably, in order to capture higher PeDs frequencies, larger samples would have had to be studied.

To date, only few studies have investigated the personality profile of patients with RBD using different methodologies, reporting conflicting results. In particular, Postuma and coll^10^, using the CMP, reported that RBD patients presented higher HA score than HCs. Similarly, several studies have described the temperament of parkinsonian patients as characterized by high HA scores^8^. On the other hand, in a 10-year prospective cohort study on iRBD patients, Postuma and coll. excluded that high HA levels at baseline could represent a risk factor for “phenoconversion” from RBD to PD^16^. Albeit this latter consideration could induce to deny the existence of a parkinsonian personality, indeed it could support the hypothesis that personality is lifelong, probably developing even earlier than RBD occurrence.

Assessing personality traits with the BFM, Baig and coll.^17^, reported higher levels of neuroticism and lower level of openess in both RBD and PD than HC. On the contrary, Sasai and coll. did not find any differences or in neuroticism level nor in openness score comparing RBD patients to HCs^18^.

To the best of our knowledge, this is the first study assessing personality profile in RBD patients strictly adhering to DSM diagnostic criteria.

Even if the cross-sectional design of our study did not allow us to define if RBD patients with OCPeD have a higher risk of *phenoconversion* respect to those without OCPeD, the high frequency of OCPeD recorded both in RBD and PD could allow us to suppose the existence of a common disease-associated RBD-PD personality profile. On this ground, considering that OCPeD has been previously observed also in never treated early PD patients^9,19^, it might be included among the pre-motor symptoms of PD.

From a pathophysiological point of view, the high prevalence of OCPeD both in PD and in RBD patients, could be related to a common fronto-striatal circuit dysfunction. Supporting this hypothesis, a resting state functional magnetic resonance imaging study reported a similar reduction in basal ganglia connectivity network in both RBD and PD patients^20^.

We are aware that some limits must be considered in interpreting our findings. The main limitation of the study is the lack of videopolisomnographic confirmation for some iRBD subjects and PD patients. However, as above specified, the diagnosis of pRBD was confirmed by an extensive clinical evaluation and a board- certified neurologist expert on sleep disorders, thus reducing the risk of false positive**.** Another limitation to consider is the relatively small sample size that could affect the statistical power of our analysis, but the strength of statistical threshold and cluster size minimize this risk. Moreover, it should be considered that iRBD is a rare disorder, with a prevalence of 5.65 for pRBD and 0.68% for dRBD^2^.

Larger prospective studies are needed confirm our data and to elucidate the predictive value of OCPeD in the early detection of patients with iRBD at risk of phenoconversion to synucleinopathies.

## Data Availability

Anonymized data will be shared as requested by qualified investigators.
